# Chemokine Networks in Blood–Brain Barrier Regulation: Bidirectional Mechanisms, Clinical Translation, and Precision Therapeutic Prospects

**DOI:** 10.3390/biom16030395

**Published:** 2026-03-05

**Authors:** Qiang Wu, Zhengjie Miao, Wen Lei, Xuewen Wu, Jingjing Zhao, Jun Sun

**Affiliations:** 1 Wuxi Medical Center, Nanjing Medical University, Wuxi 214122, China; 2024122014@stu.njmu.edu.cn (Q.W.); 2025122442@stu.njmu.edu.cn (Z.M.); katayose@stu.njmu.edu.cn (W.L.); wxw@stu.njmu.edu.cn (X.W.); 2 Department of Neurosurgery, Affiliated Wuxi People’s Hospital of Nanjing Medical University, Wuxi 214122, China

**Keywords:** blood–brain barrier, chemokines, neuroinflammation, spatiotemporal heterogeneity, therapeutic targeting

## Abstract

The blood–brain barrier (BBB), a core component of the neurovascular unit (NVU), meticulously regulates material exchange between the blood and brain parenchyma, serving as a critical barrier for maintaining the homeostasis of the central nervous system (CNS). Neuroinflammation, a pivotal response of the CNS to injury and disease, can disrupt NVU homeostasis when excessive or persistent, acting as a core pathogenic driver of various intractable neurological disorders. Chemokines, as key signaling molecules guiding the directional migration of immune cells, form the central hub mediating the dynamic regulation of neuroinflammation and the BBB. However, existing studies mostly focus on single disease systems or chemokine families, neglecting the bidirectional heterogeneity of different chemokine axes in BBB regulation and the common regulatory rules across diseases, while lacking systematic exploration of clinical translation challenges caused by the redundancy and spatiotemporal heterogeneity of the chemokine network. This review systematically clarifies the bidirectional regulatory effects of the core axes of the three major chemokine families (e.g., CCL2/CCR2, CXCL12/CXCR4, CX3CL1/CX3CR1) on the BBB. For the first time, we integrate a multi-dimensional regulatory model based on concentration, location, and time to analyze their molecular mechanisms and regulatory heterogeneity in promoting BBB disruption under pathological conditions versus mediating barrier repair and neuroprotection under specific spatiotemporal conditions. Combined with advancements in cutting-edge models such as microfluidic chips, we discuss the clinical translation progress of chemokine research, including potential biomarkers and targeted therapeutic strategies, and propose precise breakthrough paths for the two core challenges of network redundancy and spatiotemporal heterogeneity. Finally, we construct a complete research framework for chemokine-mediated regulation of NVU homeostasis, providing novel insights and directions for restoring BBB function and treating intractable neurological diseases.

## 1. Introduction

The blood–brain barrier (BBB) is the core component of the neurovascular unit (NVU). By precisely regulating material exchange between the blood and brain parenchyma, it acts as a key barrier for maintaining the internal environmental homeostasis of the central nervous system (CNS) [1]. The NVU is not a static structural complex but a dynamic functional unit composed of brain microvascular endothelial cells, tight junctions, pericytes, astrocytic endfeet, and a shared basement membrane. It can synergistically regulate cerebral blood flow, energy metabolism, and immune surveillance through complex intercellular communication, forming the structural basis for the BBB to exert its physiological functions [2]. Neuroinflammation serves as an important adaptive response of the CNS to injury and disease; moderate activation exerts a protective effect, yet excessive or persistent neuroinflammation disrupts NVU homeostasis and acts as a core pathogenic driver of various intractable neurological disorders including Alzheimer’s disease (AD), stroke, multiple sclerosis (MS), and Parkinson’s disease (PD). Under pathological conditions, the abnormal activation of microglia and astrocytes impairs BBB integrity, promoting the infiltration of peripheral immune cells such as neutrophils, monocytes, and T cells into the brain parenchyma. In turn, these infiltrating immune cells further exacerbate BBB damage and neuronal apoptosis by releasing inflammatory mediators and proteases, forming a vicious cycle between neuroinflammation and BBB disruption [3,4].

In this process, chemokines, as key signaling molecules guiding the directional migration of immune cells, play a central role as the “conductors” of immune regulation. These small-molecule cytokines, secreted jointly by NVU cells and immune cells, can precisely regulate the migratory pathways of immune cells across the BBB by forming concentration gradients, thus serving as the core hub mediating the dynamic regulation of neuroinflammation and the BBB [5,6]. Although the role of chemokines in neurological diseases has attracted extensive attention, existing relevant studies and reviews mostly focus on single disease systems or single chemokine families. They not only neglect the bidirectional heterogeneity of different chemokine axes in BBB regulation but also fail to explore the common cross-disease regulatory rules. Meanwhile, systematic discussion and solutions for the clinical translation challenges caused by the inherent redundancy and spatiotemporal heterogeneity of chemokine expression in the chemokine network are still lacking, and an integrated “mechanism–model–therapy” research framework for chemokine-mediated BBB regulation has not yet been established.

Based on this, this review systematically combs the bidirectional regulatory effects of the core axes of the three major chemokine families (CC, CXC, CX3C) on the BBB, and for the first time integrates a three-dimensional “concentration–spatiotemporal–function” regulatory model of chemokine axes. We analyze the molecular mechanisms and regulatory heterogeneity by which different axes promote BBB disruption under pathological conditions and mediate barrier repair and neuroprotection under specific spatiotemporal conditions. Combined with innovative achievements in cutting-edge models such as microfluidic chips, we discuss the clinical translation progress of chemokine research, including potential biomarkers and targeted therapeutic strategies, and propose precise breakthrough paths for the two core challenges of network redundancy and spatiotemporal heterogeneity. This review will sequentially elaborate on the bidirectional regulatory characteristics of the chemokine system, the molecular regulatory mechanisms of key axes, the core obstacles and solutions for clinical translation, and finally construct a complete research framework for chemokine-mediated regulation of NVU homeostasis, providing new ideas and directions for restoring BBB function and treating intractable neurological diseases.

## 2. The Chemokine System: A Double-Edged Sword in Blood–Brain Barrier Regulation

Chemokines are a class of small-molecule cytokines with highly conserved structures, whose core biological function is to guide the directional migration of immune cells and also serve as key regulatory molecules for immune cell transendothelial migration across the BBB during neuroinflammation [7]. According to the arrangement of conserved cysteine (C) residues at their N-terminus, chemokines are mainly divided into four subfamilies: CC, CXC, CX3C, and XC [8]. Among them, the CC, CXC, and CX3C families are the core members mediating the dynamic regulation of the BBB, and their functions all depend on specific binding to G protein-coupled receptors (GPCRs) on the cell membrane surface [9].

The chemokine signaling system exhibits significant complexity and redundancy, an important characteristic underlying its regulation of BBB function: a single receptor can be activated by multiple ligands, and a single ligand can also bind to and regulate the downstream signals of multiple receptors [10,11]. This “multi-ligand–multi-receptor” network characteristic enhances the stability of immune regulation under physiological conditions but poses enormous challenges for targeted intervention under pathological conditions. For BBB regulation, the function of chemokines is not fixed; their ultimate biological effects mainly depend on two key factors: the precise concentration gradient formed by chemokines in the BBB microenvironment, and the type and intensity of downstream signaling pathways triggered after receptor binding [12,13]. Under neuroinflammatory conditions, brain microvascular endothelial cells, astrocytes, and microglia can be abnormally activated through pathways such as NF-κB and STAT3, thereby highly expressing specific chemokines locally at the BBB and forming concentration gradients to guide the transendothelial migration of peripheral immune cells, which constitutes the molecular basis for chemokine-mediated regulation of BBB integrity [14,15].

Chemokine-mediated regulation of the BBB is not a unidirectional process of promoting disruption or protecting repair but exhibits typical bidirectional characteristics, which can be systematically explained by the three-dimensional “concentration–spatiotemporal–function” regulatory model: chemokines exert completely different biological effects at different expression concentrations, different stages of neuroinflammatory development, and different brain injury foci regions (core area/penumbra), which is the core reason for their role as a “double-edged sword” in BBB regulation. During the acute phase of neuroinflammation, in the core area of the lesion, or at high expression concentrations, chemokines mostly impair BBB integrity by recruiting proinflammatory immune cells and degrading tight junction proteins; in the reparative phase of inflammation, the penumbra of the lesion, or at low expression concentrations, their functions shift toward recruiting reparative immune cells, promoting tight junction remodeling, and mediating neuroprotection.

Notably, the core axes of different chemokine families exhibit significant heterogeneity in the intensity, trigger conditions, and regulatory logic of their bidirectional regulation: the bidirectional regulation of some axes is strictly limited by concentration thresholds and disease stages, with repair functions exerted only under specific low-concentration conditions; the functional switching of some axes is closely related to the phenotype of immune cells and is more sensitive to changes in concentration gradients; other axes exhibit the strongest bidirectionality and can achieve stable cross-disease functional switching through receptor balance and negative feedback mechanisms; there are also axes with unique neuro-immune interaction specificity, whose functional switching is directly related to glial cell polarization and molecular forms. This heterogeneity collectively constitutes the complex chemokine network regulating the BBB and determines that research and intervention cannot adopt a one-size-fits-all approach but require precise analysis combined with their inherent regulatory characteristics.

Subsequent sections of this review will conduct in-depth mechanistic exploration of the core chemokine axes of the CC, CXC, and CX3C families, systematically analyze the bidirectional effects and molecular mechanisms of each axis in regulating the BBB, clarify their concentration thresholds, spatiotemporal characteristics, and key nodes of functional switching, and provide precise molecular targets for subsequent clinical translation and targeted intervention (Table 1).

## 3. In-Depth Mechanistic Exploration of Key Chemokine Axes: Disruption and Protection

Chemokines form specific signaling axes by binding to their corresponding receptors and activate downstream pathways to precisely regulate the trans-blood–brain barrier (BBB) migration of immune cells, which constitutes the core molecular basis mediating the dynamic regulation of the BBB during neuroinflammation [16]. All chemokine axes exhibit typical bidirectional regulatory effects on the BBB: they mediate BBB disruption and amplification of neuroinflammation during the acute phase of neuroinflammation, in the core area of lesions, or at high expression concentrations, while exerting barrier repair and neuroprotective effects during the reparative phase, in the penumbral area of lesions, or at low expression concentrations. This section focuses on the core chemokine axes of the CC, CXC, and CX3C families, systematically analyzing the molecular mechanisms, concentration thresholds, and spatiotemporal characteristics underlying the bidirectional regulatory effects of each axis, and clarifying the key nodes of their functional switching combined with experimental models and data.

### 3.1. Bidirectional Regulation of the Core CC Family Axes: CCL2/CCR2 and CCL5/CCR5

CC family chemokines are the core molecules mediating the infiltration of monocytes/macrophages into the central nervous system (CNS), among which the CCL2/CCR2 and CCL5/CCR5 axes form the key signaling pathways for “monocyte/macrophage recruitment” during neuroinflammation [17]. Both axes exhibit significant spatiotemporally and concentration-dependent bidirectionality in BBB regulation, with their functional switching mainly affected by the progression stage of neuroinflammation and local microenvironmental concentration, and repair functions exerted only at the specific early reparative stage with low concentrations [18,19].

In the destructive phase, high expression concentrations in the core area of lesions during the acute phase of neuroinflammation are the core trigger conditions: in the rat middle cerebral artery occlusion (MCAO) stroke model, hemoglobin and reactive oxygen species (ROS) in the core area of lesions activate endothelial cells, astrocytes, and microglia through the NF-κB pathway, inducing their massive secretion of CCL2 (concentration > 3 ng/mL) and CCL5 (concentration > 5 ng/mL) and the formation of a high-concentration gradient locally at the BBB [20]. After CCL2 binds to CCR2 on the surface of monocytes, it recruits Ly6C-high proinflammatory monocytes to cross the BBB; these monocytes release matrix metalloproteinase 9 (MMP9) to degrade the tight junction proteins ZO-1 and Claudin-5, significantly increasing BBB permeability [21]. CCL5 binds to CCR5 and activates the JAK2/STAT3 pathway, further amplifying the tight junction degradation effect mediated by MMP9 [22]. In the subarachnoid hemorrhage model, CCL5 secreted by reactive astrocytes can directly recruit cytotoxic CD8^+^ T cells, which induce endothelial cell apoptosis by releasing perforin and granzyme B, exacerbating BBB damage [23]. In the late stage of Alzheimer’s disease (AD), the persistent stimulation of microglia by β-amyloid (Aβ) plaques leads to the high expression of CCL2/CCL5, forming a chronic high-concentration microenvironment that mediates the persistent infiltration of monocytes, ultimately aggravating BBB leakage and neuronal damage [24].

In the protective phase, low expression concentrations in the penumbral area of lesions during the subacute/chronic phase of neuroinflammation are the key to functional switching: in the chronic reparative phase of the MCAO stroke model, the expression level of CCL2 in the penumbral area of lesions drops below 1 ng/mL, and its function shifts from “recruiting proinflammatory monocytes” to “recruiting M2-type reparative macrophages”. These macrophages can phagocytose necrotic tissue, secrete transforming growth factor-β (TGF-β), and promote the proliferation of brain microvascular endothelial cells through the PI3K/Akt pathway, accelerating the reassembly of tight junction proteins and restoring BBB integrity [25,26].

When the concentration of CCL5 in the penumbral area drops below 1 ng/mL in the same period, CCL5 secreted by reactive astrocytes can activate CCR5 [27], promote the proliferation and differentiation of neural stem cells, and mediate the “moderate formation” of glial scars, which not only avoids excessive BBB leakage but also prevents the excessive proliferation of glial scars from blocking neural circuits. In the early stage of Aβ deposition in AD, low-concentration CCL2 (<0.5 ng/mL) in a humanized chip model can enhance the phagocytic capacity of microglia for Aβ plaques, limiting Aβ-induced BBB damage [28,29]. In the experimental autoimmune encephalomyelitis (EAE) model, CCL2 can guide the migration of neural stem cells to demyelinated lesions and promote remyelination by paracrine brain-derived neurotrophic factor (BDNF), indirectly maintaining the structural integrity of the BBB [30].

### 3.2. Dual Functions of the Core CXC Family Axis: CXCL8/CXCR2

In contrast to the dominant role of the CC family in monocyte recruitment, the CXCL8/CXCR2 axis is a key regulatory factor for neutrophil activation and trans-BBB migration during neuroinflammation [31,32]. Its functions exhibit obvious “spatiotemporal-concentration dual dependence”: it mediates BBB damage or neuroprotective effects according to concentration changes at different time points and different lesion locations after stroke. In addition, it exhibits unique anti-inflammatory and repair value during the remission phase of multiple sclerosis (MS) [33].

High-concentration CXCL8 is the core factor driving BBB leakage in the core area of lesions during the hyperacute phase of neuroinflammation. In the hyperacute phase (<24 h) of the MCAO stroke model, activated microglia, astrocytes, and brain microvascular endothelial cells in the core area of lesions secrete a large amount of CXCL8 through the NF-κB pathway, with the local concentration reaching > 10 ng/mL and forming a strong chemotactic gradient in the humanized chip model [34]. The high-affinity binding of CXCL8 to CXCR2 on the surface of neutrophils induces neutrophils to release MMP9 and elastase, directly degrading the BBB basement membrane and tight junction proteins; at the same time, it can directly activate the downstream signals of CXCR2 in endothelial cells, triggering the rearrangement of the actin cytoskeleton and disrupting the ordered distribution of tight junction proteins. This dual mechanism leads to a sharp increase in BBB permeability. In the acute phase of MS, astrocytes in the spinal cord and brain parenchyma lesions overexpress CXCL8, recruiting a large number of neutrophils to infiltrate the demyelinated areas. These neutrophils release inflammatory mediators such as interleukin-6 (IL-6) and tumor necrosis factor-α (TNF-α), further amplifying neuroinflammation and exacerbating BBB damage and myelin destruction [35].

Moderate and low concentrations of CXCL8 in the penumbral area of lesions during the reparative phase of neuroinflammation trigger phenotypic switching of neutrophils, thereby exerting multi-dimensional BBB protective and neuroregenerative functions. In the subacute phase of the stroke lesion penumbra, the concentration of CXCL8 drops to 2~5 ng/mL in the rat model. At this time, the recruited neutrophils undergo phenotypic switching, no longer releasing proinflammatory proteases but instead activating the ROS scavenging system to clear toxic reactive oxygen species accumulated after local ischemia, reducing the oxidative damage of endothelial cells and prolonging the survival time of neurons in the penumbra [36].

Meanwhile, CXCL8 can activate the Erk1/2 pathway in endothelial cells through CXCR2, promoting the phosphorylation and remodeling of tight junction proteins and enhancing the barrier stability of the BBB [37]. During the remission phase of MS, the maintenance of moderate and low CXCL8 concentrations (3~5 ng/mL) in human samples can regulate the binding affinity of neutrophils to the brain endothelial cell adhesion molecule ICAM-1, preventing the excessive infiltration of immune cells; at the same time, it chemotaxes oligodendrocyte precursor cells to migrate to demyelinated lesions and promotes remyelination by paracrine nerve growth factor (NGF) [35,38], indirectly maintaining the structural integrity of the BBB. In the chronic phase of stroke, the pro-angiogenic effect of CXCL8 in the ischemic area is particularly significant: it can activate endothelial cell proliferation and lumen formation, improve local cerebral blood flow perfusion, and provide nutritional support for BBB repair and neural circuit reconstruction [39].

### 3.3. Concentration-Dependent Regulation of the CXC Family Homeostatic Axis: CXCL12/CXCR4-ACKR3

CXCL12/CXCR4-ACKR3 is the chemokine axis with the most significant bidirectionality among all chemokine axes, and it also has a basal barrier maintenance function under physiological conditions. Functional switching under pathological conditions is triggered primarily by concentration, and the regulatory pattern is consistent across various neurological diseases including AD, Parkinson’s disease (PD), and stroke [40]. ACKR3, a receptor with no signal transduction function, can regulate its binding ratio with CXCR4 by endocytosing and degrading CXCL12, serving as a key negative feedback regulatory molecule for the functional balance of this axis [41].

High-concentration expression of CXCL12 during disease onset disrupts the CXCR4-ACKR3 receptor balance, thereby amplifying neuroinflammation and BBB damage. In the pathological process of PD, α-synuclein aggregation induces astrocytes to secrete CXCL12 continuously, with the local concentration rising to ≥2.5 ng/mL (humanized chip model). Activation of the CXCR4 pathway mediates the M1 polarization of microglia, releasing proinflammatory factors such as IL-1β and TNF-α to damage tight junction proteins, and simultaneously recruiting the abnormal infiltration of peripheral monocytes into the substantia nigra, exacerbating dopaminergic neuron damage and BBB leakage [42,43]. In the acute phase of stroke, ischemic necrotic cells in the core area of lesions stimulate neuroendothelial cells to produce a large amount of CXCL12, and the high-concentration gradient leads to the abnormal aggregation of peripheral monocytes around blood vessels and the formation of secondary inflammatory foci. These monocytes release proteases to degrade the BBB basement membrane, aggravating cerebral edema [44]. In the late stage of AD, hyperphosphorylated tau protein upregulates the expression of CXCL12 and simultaneously inhibits the ACKR3-mediated degradation of CXCL12, leading to the continuous activation of CXCR4 signaling, the maintenance of the proinflammatory phenotype of microglia, and the acceleration of BBB dysfunction and cognitive decline [45].

In contrast, persistent low expression under physiological conditions and moderate low expression during the disease reparative phase are the core modes for this axis to exert BBB protective and homeostatic reconstruction functions. Under physiological conditions, the binding of CXCL12 (<0.5 ng/mL in human samples) to CXCR4 can upregulate the expression of PECAM-1 in endothelial cells, enhance the stability of interendothelial junctions [13], and serve as an important molecule for maintaining the basal integrity of the BBB. In the early stage of Aβ deposition in AD, CXCL12 at a concentration of 0.5~1.0 ng/mL in the humanized chip model can promote the M2 polarization of microglia, enhance their phagocytic and clearance capacity for Aβ plaques, and reduce Aβ-induced endothelial cell damage, indirectly protecting the BBB [46]. In the penumbral area of lesions during the subacute phase of stroke, CXCL12 at a concentration of 1.0~2.0 ng/mL in the rat model regulates the balance of CXCR4-ACKR3, restricting the excessive infiltration of monocytes while promoting the proliferation of brain microvascular endothelial cells and tight junction remodeling through the PI3K/Akt pathway; at the same time, it can guide the migration of neural stem cells to the injured site, promoting neural repair and BBB reconstruction [47]. During the remission phase of MS, low-concentration CXCL12 can inhibit the adhesion of neutrophils to endothelial cells, reduce the inflammatory recurrence of demyelinated lesions, and create a stable microenvironment for remyelination [42,48].

### 3.4. Bidirectional Neuro-Immune Interaction of the CX3C Family Axis: CX3CL1/CX3CR1

The CX3CL1/CX3CR1 axis is the core pathway mediating communication between neurons and glial cells, and its bidirectional regulation has unique neuro-immune interaction specificity with no clear concentration threshold trigger [49]. Functional switching is mainly directly related to the molecular form of CX3CL1, the polarization state of microglia, and the level of pyroptosis, making it the only chemokine axis that directly integrates neuronal signals with BBB homeostasis to date. CX3CL1 is mainly expressed in a membrane-bound form on the surface of neurons and endothelial cells and can be cleaved into a soluble form by the protease ADAM17. Its cleavage efficiency is increased by 3~5 times under tau protein aggregation or lipopolysaccharide (LPS) stimulation, and the conversion of molecular forms is an important prerequisite for the functional switching of this axis [50].

When membrane-bound CX3CL1 is the dominant form in the early stage of inflammation, this axis exerts indirect BBB protection and neuronal anti-injury effects by regulating neuron-microglia communication [51]. The binding of membrane-bound CX3CL1 on the neuronal surface to CX3CR1 on the microglial surface can directly inhibit the activation of the NF-κB pathway, reduce the excessive activation of microglia, and decrease the release of proinflammatory factors such as IL-1β and TNF-α; at the same time, it can recruit microglia to clear debris around injured neurons, preventing the spread of inflammatory responses to vascular regions and reducing inflammatory invasion of the BBB from the source.

In the recovery phase of traumatic brain injury (TBI), CX3CL1 can further guide the polarization of glial cells to the M2 type, promoting the repair of vascular endothelial cells and tight junction remodeling through the secretion of factors such as TGF-β and BDNF, and accelerating the recovery of BBB function. In the subacute phase of neuroinflammation, exogenous CX3CL1 can also inhibit the activation of the NLRP3 inflammasome by reducing the phosphorylation of P65 in the NF-κB pathway, decrease the release of IL-1β and IL-18, alleviate microglial pyroptosis, and thus avoid secondary BBB damage caused by pyroptosis.

Under persistent neuroinflammation, when soluble CX3CL1 becomes the dominant form, the function of this axis is reversed and becomes an amplifying factor for BBB damage [52,53]. In the late stage of cerebral ischemia–reperfusion injury, excessive activation of the CX3CR1 signal induces microglia to release MMPs, directly degrading the BBB basement membrane and aggravating cerebral edema [50]. In the late stage of AD, tau protein tangles induce the massive cleavage of membrane-bound CX3CL1 on the neuronal surface into a soluble form. The binding of this soluble CX3CL1 to microglial CX3CR1 significantly inhibits the phagocytic function of microglia, simultaneously enhancing their release of proinflammatory factors and recruiting the infiltration of peripheral monocytes into the brain parenchyma, which doubly exacerbates BBB damage from two aspects: “decreased clearance capacity” and “inflammatory amplification” [54]. Under pathological conditions stimulated by LPS, the TLR4 pathway upregulates the expression of CX3CL1, forming a proinflammatory positive feedback loop; at the same time, CX3CR1-mediated calcium influx activates caspase-3, inducing the conversion of cells from apoptosis to pyroptosis by cleaving GSDME, further amplifying neuroinflammation and BBB disruption [55,56].

### 3.5. Heterogeneity in the Bidirectional Regulation of Each Chemokine Axis

**Figure 1:**
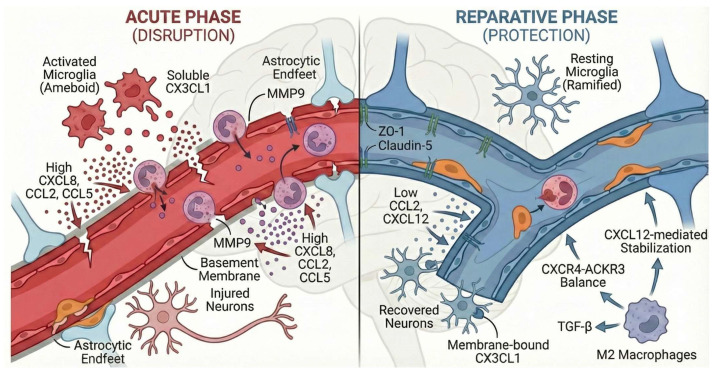
In the acute phase of neuroinflammation or in the core area of the lesion, chemokines (such as CXCL8, CCL2, CCL5) are expressed in high concentrations. These high concentrations of chemokines activate microglia to form an “angry” amoeba-like state (Ameboid Microglia) and induce the production of soluble CX3CL1. At this time, chemokines recruit a large number of pro-inflammatory immune cells (e.g., neutrophils, monocytes) and release proteases such as MMP9, leading to basement membrane degradation and destruction of tight junction proteins (ZO-1, Claudin-5), resulting in BBB leakage and neuronal damage. In the subacute/chronic phase of inflammation or in the penumbra zone of the lesion, chemokine concentrations are reduced to a specific threshold (low concentrations, such as CCL2 < 1 ng/mL). At this time, chemokine function is reversed, and M2 repair macrophages are mainly recruited. CXCL12 maintains equilibrium with CXCR4-ACKR3 receptors in the microenvironment, and membrane-bound CX3CL1 dominates, inhibiting microglial overactivation. M2 macrophages secrete factors such as TGF-β to promote vascular endothelial cell proliferation and tight junction protein remodeling, thereby repairing the BBB and exerting neuroprotective effects.

The bidirectional regulation of the BBB by the core chemokine axes of the CC, CXC, and CX3C families during neuroinflammation does not follow a unified regulatory logic but exhibits significant heterogeneity. Each axis has obvious differences in the intensity of bidirectional regulation, trigger conditions for functional switching, and sensitivity to network redundancy. This heterogeneity collectively constitutes a complex but ordered chemokine regulatory network during neuroinflammation (Figure 1).

The CCL2/CCR2 and CCL5/CCR5 axes exhibit weak bidirectionality; their functions are strictly limited by the progression stage of the disease and the concentration of the local microenvironment, and their barrier repair capacity is only manifested in the early stage of the disease with low concentrations. Meanwhile, they are easily interfered by the redundancy effect of the chemokine network, and intervention of a single pathway is difficult to achieve stable regulatory effects. The CXCL8/CXCR2 axis has stable and significant bidirectionality; its functional switching mainly depends on changes in concentration gradients and phenotypic switching of neutrophils, with low sensitivity to the redundancy of the chemokine network, making it a pathway for which precise regulation is relatively easy to achieve in targeted intervention. The CXCL12/CXCR4-ACKR3 axis is the chemokine axis with the strongest bidirectionality; it achieves stable cross-disease functional plasticity through concentration-dependent receptor binding balance and ACKR3-mediated negative feedback mechanisms, and its regulatory pattern is consistent across various neurological diseases such as AD, PD, stroke, and MS, serving as the core regulatory node in the chemokine network. The CX3CL1/CX3CR1 axis has unique neuro-immune interaction specificity; its functional switching is directly related to the molecular form of CX3CL1, the polarization state of microglia, and the level of pyroptosis, and it is also the only pathway that can directly integrate neuronal signals with BBB homeostasis. The core of its regulation lies in maintaining the normal communication between neurons and glial cells.

## 4. Core Challenges and Coping Strategies for Chemokine-Mediated Blood–Brain Barrier Regulation

The ultimate goal of research on the role of the chemokine network in the dynamic regulation of the BBB is to achieve clinical translation to guide the precision treatment of neurological diseases. However, this process is restricted by the biological characteristics of chemokines themselves and faces two core challenges: network redundancy and spatiotemporal heterogeneity. Meanwhile, the limitations of traditional research models have also become an important obstacle hindering the connection between mechanistic analysis and clinical translation. In response to the above problems, the research field is seeking breakthroughs through experimental model innovation, exploration of specific biomarkers, and development of multi-dimensional therapeutic strategies, forming a complete research logic of “problem discovery-problem solving-clinical translation”, which provides key support for breaking the clinical translation bottleneck of chemokine-mediated BBB regulation.

### 4.1. Core Challenges: Redundancy and Spatiotemporal Heterogeneity of the Chemokine Network

The fundamental reason why the regulatory effect of chemokines on the BBB is difficult to translate into clinical practice lies in the inherent network redundancy and spatiotemporal heterogeneity of chemokines, which are intertwined, making it difficult for a single mechanistic study and targeted intervention strategy to achieve the desired effect.

Chemokines and their receptors form a highly redundant signaling network through a “many-to-many” binding mode. Although this characteristic guarantees the body’s immune homeostasis under physiological conditions, it becomes a therapeutic challenge under pathological conditions. On the one hand, chemokines from different families can synergistically amplify neuroinflammation by sharing receptors or signaling pathways. For example, in AD, platelet-derived CCL5 and CX3CL1 can jointly promote the polarization of glial cells to a proinflammatory phenotype and recruit monocytes, accelerating the pathological process; in PD, the co-expression of CCR5 and CX3CR1 on peripheral T cells synergistically promotes their migration to the substantia nigra, leading to the loss of dopaminergic neurons. Inhibition of CCL5 alone cannot block Aβ deposition and neuroinflammation mediated by CX3CL1 through CX3CR1, which greatly limits the efficacy of single-target antagonists.

On the other hand, some chemokines can exert antagonistic effects through competitive co-receptor binding. For example, in a mouse model of tau pathology, CXCL12 can competitively inhibit the CCR5-mediated proinflammatory signal in microglia, and the CX3CL1-CX3CR1 axis can inhibit CCR2-driven macrophage infiltration and promote their conversion to an anti-inflammatory phenotype. Such complex synergistic and antagonistic relationships make single-target intervention prone to induce the activation of compensatory parallel pathways, making it difficult to achieve effective regulation of the chemokine network.

The function of chemokines is strictly dependent on their spatiotemporal expression pattern, a characteristic that is particularly prominent in ischemic stroke and also breaks the homogeneous understanding of the brain microenvironment. From the temporal dimension, the acute phase (24~72 h) after stroke is dominated by proinflammatory chemokines such as CCL2 and CXCL8, which massively recruit neutrophils and monocytes and damage BBB integrity; the chronic phase is dominated by reparative chemokines such as CXCL12, which participate in glial scar formation and tissue remodeling.

From the spatial dimension, the ischemic core undergoes rapid necrosis accompanied by a severe inflammatory response with high-concentration chemokine expression, while the release of chemokines in the surrounding penumbra is delayed and low-intensity, forming an important therapeutic window; without timely intervention, the penumbra will gradually transform into the ischemic core under the action of persistent low-grade inflammation mediated by CCL5 and other factors.

**Figure 2:**
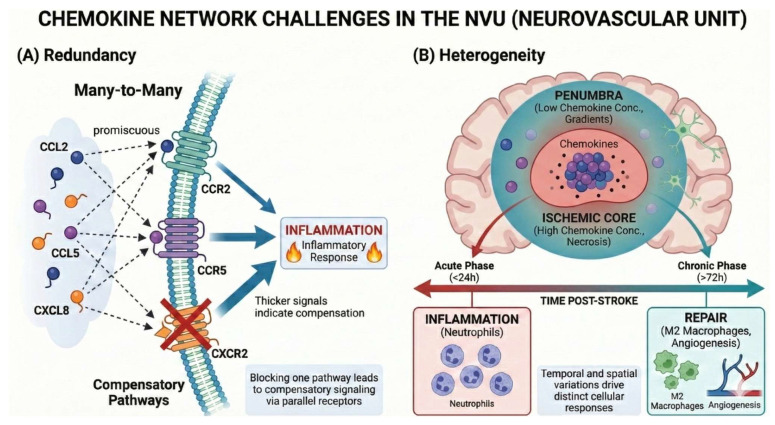
(**A**) There is a high degree of “many-to-many” redundancy between chemokine ligands and receptors, and blockade of a single receptor (e.g., CXCR2) often induces compensatory activation of parallel signaling pathways (e.g., CCR2/CCR5), leading to persistent inflammatory responses. (**B**) The regulation of chemokines on the blood–brain barrier (BBB) showed significant heterogeneity: in the ischemic core region and the acute phase (<24 h), high concentrations of chemokines mainly recruited inflammatory neutrophils and caused BBB destruction; while in the chronic phase (>72 h) and the penumbra zone region, the low-concentration gradient microenvironment promoted the immune response to repair phenotype, mediating M2 macrophage recruitment and angiogenesis. This complex dynamic network characteristic suggests that it is difficult to inhibit inflammation or single-target intervention alone, and precise regulation strategies with spatiotemporal specificity need to be developed.

This spatiotemporal heterogeneity requires that the targeted intervention of chemokines must have stage-specificity and regional-specificity, and the traditional one-size-fits-all therapeutic approach is obviously unable to meet this demand, further increasing the difficulty of clinical translation (Figure 2).

### 4.2. Innovation of Advanced Experimental Models—Unique Advantages and Applications of BBB Chips

Analyzing the redundancy and spatiotemporal heterogeneity of the chemokine network first requires research models that can closely simulate the human brain microenvironment. Traditional research models are difficult to reproduce the physiological and pathological characteristics of the BBB, while microfluidic BBB chips, as a new type of organoid model, offer distinct advantages in addressing specific biological problems in the study of chemokine-mediated BBB regulation compared with traditional in vitro models and in vivo animal models.

Compared with traditional in vitro models, the Transwell co-culture system can only simulate the basic intercellular interactions and chemokine gradients but cannot reproduce the key physiological characteristics of the BBB such as blood flow shear stress and three-dimensional spatial structure, making it difficult to accurately reproduce the real process of chemokine-guided immune cell trans-BBB migration [57,58]. In contrast, the BBB chip adopts a complex “sandwich” structure, generates physiologically relevant fluid shear stress through micropumps, and integrates brain microvascular endothelial cells, pericytes, and astrocytes to construct a biomimetic neurovascular unit (NVU) microenvironment. It can accurately construct and maintain the concentration gradient of chemokines, recapitulating key aspects of the process of chemokine-guided directional migration of immune cells in vivo.

However, it must be acknowledged that cells isolated from tissue and cultured in vitro may lose specific phenotypic characteristics due to the absence of the complete systemic environment found in vivo. While in vitro models cannot fully replace the systemic complexity of animal research, the BBB chip can provide a more human physiology-relevant in vitro screening platform for chemokine-targeted drugs, enabling high-throughput drug screening and greatly improving the efficiency of drug research and development [59,60]. For example, the screening of CXCR2 inhibitors based on BBB chips constructed with human induced pluripotent stem cells (iPSC) yields IC50 values that correlate better with human responses than those of the traditional Transwell model, and the screening results are more referenceable [61].

Compared with in vivo animal models (such as the MCAO model and EAE model), the BBB chip can mitigate the problems of species differences and ethical disputes [62], solving the core biological problem of low accuracy of animal models in predicting clinical efficacy. Moreover, species differences in animal models lead to significant differences in chemokine concentration thresholds and receptor binding efficiency from humans, making it difficult to directly translate research results into clinical practice. At present, the BBB chip has initially established international cell seeding standards, and neural cells differentiated from iPSCs are used to replace immortalized cell lines, further improving the humanized simulation effect and enabling more accurate reproduction of chemokine expression patterns and BBB regulatory rules in human neurological diseases.

In addition, in response to the biological problem of dynamic changes in chemokines during chronic neuroinflammation, although the stable cell function time of the current BBB chip is only 1~2 weeks with certain limitations, compared with the defect of traditional in vitro models that cannot simulate chronic inflammation, the research field is continuously improving through optimizing cell sources and establishing special BBB chip standards, gradually improve the simulation of the dynamic regulatory process of chemokines under chronic inflammation [63,64,65].

At present, the BBB chip has successfully simulated the process of CXCL8-mediated neutrophil trans-BBB migration, with a correlation coefficient of 0.91 between its migration rate and the in vivo data of MCAO model mice, fully verifying its reliability in chemokine mechanism research. This model serves as a valuable complementary tool for analyzing the spatiotemporal heterogeneity and network redundancy of chemokines, bridging the gap between mechanistic research and clinical translation (Table 2).

### 4.3. Exploration of Biomarkers and Their Clinical Translation Value

In response to the complexity of the chemokine network, exploring biomarkers that can accurately reflect the degree of BBB damage and the state of neuroinflammation is the key to achieving early clinical diagnosis and prognostic evaluation, and also provides potential intervention targets for targeted therapy. The levels of chemokines and related inflammatory mediators in cerebrospinal fluid and peripheral blood are closely related to BBB permeability, becoming the core direction for biomarker exploration, among which soluble cluster of differentiation 40 ligand (sCD40L) is one of the most maturely studied biomarkers with the highest clinical translation potential to date.

Clinical studies have found that the expression level of sCD40L is highly correlated with the pathological process of neurological diseases: the increase in sCD40L in patients with mild stroke or transient ischemic attack (TIA) is significantly associated with the risk of stroke recurrence, and abnormally high expression of sCD40L has also been detected in the cerebrospinal fluid of patients with MS and neuromyelitis optica spectrum disorders (NMOSD) [66,67]. Subsequent in vitro and animal model studies have further clarified its mechanism of action: sCD40L can activate the CD40/CD40L signaling pathway, induce the high expression of adhesion molecules such as ICAM-1 and vascular cell adhesion molecule 1 (VCAM-1) in endothelial cells, thereby promoting immune cell infiltration and disrupting BBB integrity [68]. This mechanism indicates that sCD40L is not a simple disease-associated biomarker but directly participates in the pathological process of BBB damage, endowing it with dual clinical value as both a predictive index and a therapeutic target.

In the future, the detection of a single chemokine or inflammatory mediator can no longer meet the needs of clinical precise diagnosis. Combining multi-omics data (proteomics, metabolomics) to establish a multi-biomarker prediction model integrating sCD40L, specific chemokine combinations, cerebrospinal fluid biochemical indicators, etc., will become the development trend. This model can more accurately evaluate the state of BBB damage and the level of neuroinflammation, provide diagnostic basis for subsequent personalized targeted therapy, and realize the leap from “single biomarker detection” to “multi-dimensional precise classification”.

### 4.4. Development of Therapeutic Strategies Targeting the Chemokine Network

In response to the characteristics of redundancy and spatiotemporal heterogeneity of the chemokine network, traditional single-target intervention strategies can no longer meet the clinical treatment needs of CNS diseases. At present, the research field is developing chemokine network-targeted therapeutic strategies from three core directions: multi-target antagonism, precise targeted delivery, and stage-specific regulation. Through multi-dimensional synergistic intervention, effective regulation of the chemokine network is achieved. Meanwhile, the efficacy and safety verification are carried out in combination with advanced functional models such as BBB chips, providing experimental support for improving the clinical translation efficiency of therapeutic strategies.

The “many-to-many” binding mode between chemokines and receptors is the key reason why single-target intervention is prone to induce compensatory pathway activation. Multi-target antagonists have become the core technical path to solve the redundancy of the chemokine network by blocking multiple key chemokine axes simultaneously. At present, a variety of multi-target antagonists have entered the stage of basic research and clinical exploration. For example, Cenicriviroc, a CCR2/5 dual-target antagonist, and Reparixin, a CXCR1/2 dual-target antagonist, can enhance the regulatory efficacy on complex neuroinflammation by synchronously blocking multiple proinflammatory chemokine axes [69,70]. In addition, blocking the release of multiple chemokines from the source is also an important idea to solve network redundancy: GPVI antagonists can reduce the release of multiple proinflammatory chemokines such as CCL2, CCL5, and CXCL8 by platelets by inhibiting platelet activation, blocking the synergistic redundant effect of chemokines at the upstream [71]; the combined application of anti-CCL2 monoclonal antibody and anti-CXCL8 monoclonal antibody can synchronously reduce the central infiltration of monocytes and neutrophils, avoiding the compensatory increase in other chemokines after single ligand neutralization, and has shown significant BBB protective effects in MS and post-ischemic neuroinflammation models [35,72].

Systemic administration is prone to interfere with the body’s normal immune surveillance function, and biological agents such as chemokine antagonists and small-molecule inhibitors are difficult to cross the BBB to reach the target area in the brain, which has become a key obstacle restricting the clinical translation of chemokine-targeted therapy. Precision drug delivery systems (DDS) based on nanotechnology can achieve efficient trans-BBB delivery of drugs and targeted enrichment in brain lesion areas to a certain extent through specific modification of carriers, providing a feasible solution for solving the spatiotemporal heterogeneity problem of chemokine intervention.

At present, in addition to transferrin receptor (TfR) antibody-modified liposomal nanoparticles (the brain drug concentration can be increased by 8 times after loading CXCR1/2 inhibitors, and the side effects related to peripheral immune suppression can be reduced), ligand-mediated vascular immune targeting strategy has become a research hotspot in CNS targeted delivery [73,74]. Relying on the precise design of endothelial cell adhesion molecules, this strategy achieves efficient enrichment of DDS in the CNS: DDS targeting PECAM, ICAM, and VCAM has been verified by isotope tracing to have a brain enrichment efficiency at least 10 times higher than that of TfR ligand-mediated delivery systems [75]; the injection of such targeted preparations via the carotid artery or cerebral artery can further enhance the drug uptake efficiency of the CNS, and in animal models with different genetic backgrounds such as rats, pigs, and mice, the enrichment amount of such preparations in the ipsilateral cerebral hemisphere of the injury is 4~10 times that of the contralateral side [76].

Among them, VCAM ligand-mediated DDS can effectively bypass the pulmonary vascular system, and its enrichment efficiency and specificity to brain endothelium are increased by an order of magnitude compared with PECAM and ICAM targeted DDS; ICAM targeted DDS can achieve the transport to marginal leukocytes through pulmonary microvascular endothelium in the acute brain injury mouse model, a process closely related to the transmission of injury signals by cytokines and chemokines released from the damaged CNS to the lungs through the blood circulation, providing a new direction for the delivery strategy of cross-organ regulation of CNS inflammation [77]. In addition, intelligent nanocarriers loaded with BBB protectants such as claudin-5 enhancers and RepSox can achieve local efficient release of drugs in target areas such as the ischemic penumbra, initially meeting the needs of stage-specific and regional-specific treatment [73,74].

In the future, the core development direction of chemokine-targeted therapy is to transform from the traditional one-size-fits-all intervention mode to a precise neuro-immunotherapy mode. The realization of this strategy needs to be based on multi-omics analysis technology and simultaneously achieve precise regulation of the location, timing, amplitude, and duration of drug delivery. Specifically, through proteomic and transcriptomic detection of patients’ blood and cerebrospinal fluid samples, the unique chemokine network dysregulation pattern of each individual can be clarified, and then personalized classification treatment can be carried out.

For example, AD patients are divided into “CCL2 high-expression type” and “CXCL12 imbalance type”, and CCR2 inhibitors and ACKR3 agonists are used for targeted intervention respectively; in response to the dynamic differences in chemokine expression between the acute and chronic phases of stroke, stage-specific treatment regimens of “BBB stabilization emergency intervention” and “proinflammatory-anti-inflammatory/repair signal balance regulation” are implemented respectively [78,79]. In the verification of treatment regimens, humanized BBB chips can simulate patient-specific chemokine concentrations (such as chemokine mixtures derived from the cerebrospinal fluid of AD patients), providing a high-throughput verification platform for personalized combination treatment regimens, but the detection results still need to be further verified in combination with in vivo experiments.

Although in vitro models can provide key data at the cellular and molecular biological levels, their microenvironment is different from the in vivo physiological/pathological state, making it difficult to completely reproduce the phenotypic characteristics and tissue composition of the cerebrovascular compartment. Therefore, it is necessary to objectively combine the high-throughput advantages of in vitro models with the physiological relevance of in vivo models to carry out multi-dimensional verification. This synergistic strategy of “classified diagnosis-precise delivery-multi-model verification” can shorten the clinical translation cycle of chemokine-targeted therapy and provide support for improving the clinical application potential of therapeutic strategies.

## 5. Perspective

However, the current research on chemokine network-mediated BBB regulation still has many unsolved limitations, which have become key barriers to the translation of basic research into clinical practice.

First, there are technical shortcomings in model research: although the BBB chip has achieved humanized bionics of the NVU, it still faces problems such as insufficient standardization of cell sources, limited long-term culture stability (only maintaining for 1-2 weeks), and lack of simulation of in situ neuron-immune cell interaction; although animal models can reproduce the overall pathological environment, there are species differences in chemokine receptor binding affinity and signaling pathway activation efficiency, making it difficult to accurately predict clinical efficacy.

Second, there is a significant gap in human data for mechanistic research: the existing chemokine concentration thresholds and spatiotemporal regulatory rules are mostly based on in vitro chips and animal models (MCAO, EAE, etc.), with a lack of direct detection data of local chemokines in the human brain, and the human pathological evidence for the functional switching of chemokine axes in chronic neurological diseases such as AD and PD is still insufficient.

Third, therapeutic strategies are still in the early exploratory stage: single-target antagonists are limited in efficacy due to the redundancy of the chemokine network, the long-term safety of multi-target antagonists has not been systematically verified, and systemic administration is prone to disrupt peripheral normal immune surveillance; meanwhile, the research and development of targeted delivery systems that can efficiently cross the BBB are immature, becoming a core obstacle to the clinical application of biological agents and small-molecule inhibitors.

Fourth, the technical support for clinical precise classification is insufficient: chemokine network classification based on multi-omics has not yet been clinically popularized, and in response to the spatiotemporal heterogeneity of BBB regulation, there is a lack of precise tools for real-time in vivo monitoring, making it difficult to achieve individualized regulation of different disease stages and brain regions.

In response to the above challenges, the clinical translation of chemokine network-mediated BBB regulation should follow a phased and progressive research and development path, relying on the step-by-step advancement of "mechanistic analysis–model optimization–clinical verification" to realize the transformation from basic research to clinical application.

In the short term (1-3 years), the focus is on the clinical verification and standardized detection of biomarkers, expanding the clinical sample size of potential biomarkers such as sCD40L, clarifying their diagnostic and prognostic thresholds in diseases such as stroke, MS, and AD, and developing a combined "blood + cerebrospinal fluid" detection kit to achieve rapid assessment of BBB damage and neuroinflammation levels; meanwhile, improve the standardization system of BBB chips, establish core parameters such as cell seeding and fluid shear stress dedicated to neuroscience, and enhance their reliability and repeatability in drug screening.

In the medium term (3-5 years), promote the precise classification of patients based on multi-omics (such as proteomics and transcriptomics), construct chemokine network classification panels for AD and stroke (such as CCL2 high-expression type and CXCL12 imbalance type) to achieve precise division of patient subgroups; carry out high-throughput screening of multi-target antagonists and verification of combination drug regimens relying on the optimized humanized BBB chip, and simultaneously develop nanoscale targeted delivery systems such as TfR antibody modification to improve the brain delivery efficiency of drugs and reduce peripheral side effects.

In the long term (5-8 years), carry out small-scale clinical basket trials based on chemokine classification to evaluate the improvement effect of multi-target antagonists combined with targeted delivery systems on BBB integrity and neurological function, and accumulate clinical efficacy and safety data; meanwhile, establish a long-term follow-up database for patients, iteratively optimize the drug dosage and treatment timing according to the chemokine expression characteristics of different disease stages, and realize stage-specific and regional-specific regulation of neuroinflammation [80,81].

## 6. Conclusions

Chemokine-mediated intercellular communication is the core hub for the dynamic regulation of blood–brain barrier (BBB) function. It is not a simple binary regulatory mode of "activation/inhibition" but forms a bidirectional dynamic balance system highly dependent on concentration and spatiotemporal characteristics. This review systematically clarifies the dual damaging and protective effects of the three major chemokine families (CC, CXC, CX3C) on the BBB during neuroinflammation through complex receptor networks: on the one hand, they can disrupt BBB integrity by recruiting immune cells and degrading tight junction proteins, exacerbating the neuroinflammatory cascade reaction; on the other hand, under specific concentration and spatiotemporal microenvironments, they can mobilize reparative cell populations, promote vascular endothelial remodeling and neural tissue repair, and maintain BBB homeostasis.

The bidirectional regulation of different chemokine axes exhibits significant heterogeneity: the CXCL12/CXCR4-ACKR3 axis has the strongest functional plasticity, while the CX3CL1/CX3CR1 axis realizes the direct coupling of neuronal signals with BBB homeostasis. The three-dimensional "concentration–spatiotemporal–function" regulatory model constructed based on this provides a core framework for analyzing the regulatory rules of the chemokine network. Meanwhile, the research and application of advanced models such as microfluidic BBB chips have broken the technical bottlenecks of traditional in vitro and animal models, realizing the precise analysis of chemokine regulatory mechanisms and efficient screening of targeted drugs. The preliminary exploration of biomarkers such as sCD40L and single/multi-target chemokine antagonists has also laid a foundation for the clinical intervention of BBB-related neurological diseases, forming a complete "mechanism–model–therapy" research system as a whole and providing novel insights for the restoration of neurovascular unit (NVU) homeostasis.

Targeting the chemokine network to restore BBB homeostasis is a highly promising dynamic regulatory strategy for the treatment of intractable neurological diseases such as stroke, AD, and MS. Its core is not the indiscriminate inhibition of neuroinflammation but the realization of the balance of "inhibiting pathological inflammation and activating reparative immunity" through the precise regulation of the bidirectional functions of the chemokine network. The key to future research lies in the in-depth integration of multi-omics-driven precise molecular classification, nanotechnology-mediated spatiotemporally specific targeted delivery, and highly biomimetic advanced disease models, breaking the dual challenges of chemokine network redundancy and spatiotemporal heterogeneity, and improving the closed-loop research system of "mechanism–model–therapy". Although many technical and clinical bottlenecks still exist at present, with the continuous optimization of advanced models and the development of precision medicine technology, the chemokine network is expected to become a key target for breaking the treatment bottleneck of intractable neurological diseases, promoting the leap of NVU homeostasis regulation from basic research to clinical precise application, and opening up a new direction for the immunotherapy of neurological diseases.

## Figures and Tables

**Table 1:** Core Characteristics of Major Chemokine Axes Regulating Blood–Brain Barrier (BBB).

Chemokine Axis	Molecular Features	Concentration Ranges (Reference Value/Threshold)	Physiological Effects	Pathological Effects (Damage/Repair)
CCL2/CCR2	Ligand MW ≈ 13 kDa; Receptor CCR2 (GPCR)	Physiological: Plasma < 0.1 ng/mL, CSF < 0.05 ng/mL; Pathological: Damage (>3 ng/mL, rat MCAO), Repair (<1 ng/mL, rat MCAO)	Maintain monocyte homeostasis; Mild immune surveillance	Damage: Recruit Ly6C^+^ pro-inflammatory monocytes, release MMP9 to degrade ZO-1/Claudin-5; Aggravate BBB leakage (stroke/late AD); Repair: Recruit M2 macrophages, secrete TGF-β; Activate PI3K/Akt pathway for endothelial proliferation and tight junction reassembly (chronic stroke)
CCL5/CCR5	Ligand MW ≈ 8 kDa; Receptor CCR5 (GPCR)	Physiological: Plasma < 0.2 ng/mL, CSF < 0.08 ng/mL; Pathological: Damage (>5 ng/mL, rat MCAO/AD), Repair (<1 ng/mL, rat MCAO)	Regulate T cell homeostasis; Mild inflammation inhibition	Damage: Activate JAK2/STAT3 pathway to amplify MMP9 effects; Recruit CD8^+^ T cells to induce endothelial apoptosis (subarachnoid hemorrhage); Repair: Promote neural stem cell proliferation/differentiation; Mediate “moderate glial scar formation” (subacute stroke); Enhance Aβ phagocytosis (early AD)
CXCL8/CXCR2	Ligand MW ≈ 8 kDa; Receptor CXCR2 (GPCR)	Physiological: Plasma < 0.3 ng/mL, CSF < 0.1 ng/mL; Pathological: Damage (>10 ng/mL, humanized chip), Repair (2~5 ng/mL, rat)	Neutrophil homeostasis; Local anti-infection	Damage: Induce neutrophils to release MMP9/elastase, degrade basement membrane; Trigger endothelial actin cytoskeleton rearrangement (hyperacute stroke); Repair: Activate ROS scavenging system; Promote Erk1/2-mediated tight junction remodeling; Chemotax oligodendrocyte precursor cells (MS remission)
CXCL12/CXCR4-ACKR3	Ligand MW ≈ 8 kDa; Receptors CXCR4 (GPCR) + ACKR3 (non-signaling)	Physiological: Plasma < 0.5 ng/mL, CSF < 0.2 ng/mL; Pathological: Damage (≥2.5 ng/mL, humanized chip), Repair (0.5~2.0 ng/mL)	Upregulate endothelial PECAM-1; Maintain BBB integrity; Stem cell homing	Damage: Mediate microglial M1 polarization to release IL-1β/TNF-α; Induce abnormal monocyte aggregation (PD/stroke); Repair: Promote microglial M2 polarization for Aβ clearance; Activate PI3K/Akt pathway for tight junction repair; Guide neural stem cell migration (subacute stroke)
CX3CL1/CX3CR1	Ligand MW ≈ 95 kDa (membrane-bound)/10 kDa (soluble); Receptor CX3CR1 (GPCR)	Physiological: Plasma < 0.15 ng/mL, CSF < 0.06 ng/mL; Pathological: Damage (increased soluble fraction, AD/ischemia–reperfusion), Repair (membrane-bound dominant)	Inhibit microglial overactivation; Neuron-glia communication	Damage: Soluble form inhibits microglial phagocytosis; Activate caspase-3-induced pyroptosis; Recruit monocytes (late AD); Repair: Membrane-bound form inhibits NF-κB pathway; Guide microglial M2 polarization to secrete TGF-β/BDNF; Inhibit NLRP3 inflammasome (TBI recovery)

**Table 2:** Comparison of Advantages and Disadvantages of Models for Chemokine-BBB Regulation Research.

Research Model	Advantages	Limitations	Application Scenarios
Transwell co-culture system(in vitro)	Simple operation, low cost; Simulate basic cell–cell interactions and chemokine gradients; High-throughput preliminary screening	Lack blood flow shear stress and 3D NVU structure; Fail to reproduce immune cell trans-BBB migration; Low biomimetic degree	Detection of basal chemokine expression; Verification of single cell–cell interaction; Preliminary drug toxicity screening
Microfluidic BBB-on-a-chip(in vitro)	Integrate endothelial cells/pericytes/astrocytes to construct biomimetic NVU; Generate physiological shear stress; Real-time visualization of immune cell migration; Humanized (iPSC-derived cells); High-throughput drug screening	Non-standardized cell sources; Limited long-term stability (1~2 weeks); Unable to simulate systemic factors; High equipment cost	Mechanism research of chemokine gradient regulation; Dynamic observation of immune cell trans-BBB migration; High-throughput screening of targeted drugs (e.g., CXCR2 inhibitors)
Animal disease models: MCAO stroke/EAE/MS/AD transgenic mice(in vivo)	Provide complete systemic environment; Simulate spatiotemporal heterogeneity of disease progression; Verify in vivo drug efficacy and safety	Significant species differences (chemokine receptor affinity/concentration threshold); Ethical controversies; Complex operation, high cost	Verification of overall effects of chemokine axes in diseases; In vivo drug efficacy and toxicity evaluation; Research on multi-organ interaction mechanisms
Gene-edited models: *CXCR4* knockout mice/CCL2 overexpressing mice(in vivo)	Specific verification of single chemokine axis function; Eliminate redundant pathway interference; Precise mechanism analysis	Long construction cycle, high cost; Potential compensatory pathway activation; Phenotypic differences from natural diseases	Verification of causal relationship of single chemokine axis; Research on key signaling pathways (e.g., NF-κB/PI3K)
Chip-animal combined model: Chip pre-screening + animal verification	Integrate high-throughput advantage of chips and physiological relevance of animals; Reduce animal usage; Improve clinical translation efficiency	Complex operation process; Need for cross-platform technical integration; Need for data consistency verification	Personalized drug screening oriented to precision medicine; Verification of complex chemokine network intervention strategies

## Data Availability

No new data were created or analyzed in this study. Data sharing is not applicable to this article.

## Abbreviations

The following abbreviations are used in this manuscript:

BBB	Blood–Brain Barrier
NVU	Neurovascular Unit
CNS	Central Nervous System
GPCRs	G Protein-Coupled Receptors
MCAO	Middle Cerebral Artery Occlusion
MMP9	Matrix Metalloproteinase 9
AD	Alzheimer’s Disease
Aβ	β-Amyloid
TGF-β	Transforming Growth Factor-β
EAE	Experimental Autoimmune Encephalomyelitis
BDNF	Brain-Derived Neurotrophic Factor
MS	Multiple Sclerosis
IL-6	Interleukin-6
TNF-α	Tumor Necrosis Factor-α
ROS	Reactive Oxygen Species
NGF	Nerve Growth Factor
PD	Parkinson’s Disease
LPS	Lipopolysaccharide
TBI	Traumatic Brain Injury
GSDME	Growth Arrest and DNA Damage-Inducible Protein E
iPSC	human Induced Pluripotent Stem Cells
IC50	Half Maximal Inhibitory Concentration
sCD40L	soluble Cluster of Differentiation 40 Ligand
TIA	Transient Ischemic Attack
NMOSD	Neuromyelitis Optica Spectrum Disorders
ICAM-1	Intercellular Adhesion Molecule 1
VCAM-1	Vascular Cell Adhesion Molecule 1
DDS	Drug Delivery System
TfR	Transferrin Receptor
GPVI	Glycoprotein VI
